# Research on Rolling Bearing Fault Diagnosis Based on Digital Twin Data and Improved ConvNext

**DOI:** 10.3390/s23115334

**Published:** 2023-06-05

**Authors:** Chao Zhang, Feifan Qin, Wentao Zhao, Jianjun Li, Tongtong Liu

**Affiliations:** 1 College of Mechanical Engineering, Inner Mongolia University of Science and Technology, Baotou 014010, China; 2 Inner Mongolia Key Laboratory for Intelligent Diagnosis and Control of Electromechanical Systems, Baotou 014010, China; 3 College of Information Engineering, Inner Mongolia University of Science and Technology, Baotou 014010, China

**Keywords:** rolling bearing, digital twin, transfer learning, ConvNext, fault detection

## Abstract

This article introduces a novel framework for diagnosing faults in rolling bearings. The framework combines digital twin data, transfer learning theory, and an enhanced ConvNext deep learning network model. Its purpose is to address the challenges posed by the limited actual fault data density and inadequate result accuracy in existing research on the detection of rolling bearing faults in rotating mechanical equipment. To begin with, the operational rolling bearing is represented in the digital realm through the utilization of a digital twin model. The simulation data produced by this twin model replace traditional experimental data, effectively creating a substantial volume of well-balanced simulated datasets. Next, improvements are made to the ConvNext network by incorporating an unparameterized attention module called the Similarity Attention Module (SimAM) and an efficient channel attention feature referred to as the Efficient Channel Attention Network (ECA). These enhancements serve to augment the network’s capability for extracting features. Subsequently, the enhanced network model is trained using the source domain dataset. Simultaneously, the trained model is transferred to the target domain bearing using transfer learning techniques. This transfer learning process enables the accurate fault diagnosis of the main bearing to be achieved. Finally, the proposed method’s feasibility is validated, and a comparative analysis is conducted in comparison with similar approaches. The comparative study demonstrates that the proposed method effectively addresses the issue of low mechanical equipment fault data density, leading to improved accuracy in fault detection and classification, along with a certain level of robustness.

## 1. Introduction

With the continuous advancement of the manufacturing industry, China’s transition from being a manufacturing power to becoming a manufacturing juggernaut has emerged as a significant task for the nation’s economic progress in the modern era [1]. Within the realm of industry, rolling bearings find extensive utilization across various apparatuses and machinery. Whenever a malfunction arises, it typically gives rise to a sequence of intricate, dynamic, and noise-obscured vibration signals, rendering the extraction of fault-related information a challenging task [2].

With the proliferation of monitoring devices and the escalation in sampling frequency, the domain of bearing fault monitoring has stepped into the realm of “big data”. Consequently, the fusion of monitoring data with artificial intelligence for fault diagnosis has become a focal point of research. Hu et al. [3] have developed an enhanced three-layer Laplace wavelet convolutional neural network that not only elucidates its physical implications but also enhances the network’s interpretability. This network exhibits a notable degree of accuracy and generalization across different types of bearing fault scenarios. However, in real-world industrial environments, the scarcity of high-quality training data for intelligent diagnostic models poses a challenge due to the transient nature of fault incidents during the prolonged normal operation of rotating mechanical equipment [4]. Furthermore, existing deep learning algorithms necessitate an extensive analysis of sample data to yield a high-performance algorithmic model. To address these concerns, Xu et al. [5] have proposed a ViT (Vision Transformer) model that leverages multi-information fusion, enabling bearing fault diagnosis with limited data samples. Additionally, Chen et al. [6] have introduced a conditional depth convolution countermeasure generation networks (C-DCGAN) model capable of enhancing small-sample, multi-category data. The vibration signals emanating from bearings in mechanical equipment exhibit characteristics of both mechanical big data and low data density. Moreover, due to their prolonged operational lifetimes in normal working conditions, the monitoring data collected often suffer from high redundancy and low data value density. In this context, the advent of the digital twin (DT) concept provides a viable solution to the aforementioned challenges [7].

The DT represents a novel technological advancement rooted in computer modeling and simulation techniques. It intricately intertwines physical systems with virtual realms, leveraging digits and information to manifest the behaviors of both real and virtual environments [8]. By employing data acquired from sensors and generated within the virtual space, the DT technology captures the present state of a system, constructs precise digital models, and conducts real-time simulations and optimizations through computers. The rapid progress of information technology, particularly the emergence of next-generation technologies such as industrial IoT, cloud computing, big data, and machine learning, has propelled DT technology into the forefront of industrial research [9,10,11,12]. The inception of the DT concept can be traced back to Professor Michael Grieves’ 2003 proposal at the University of Michigan in the United States [13]. Initially, DT technology found applications in the military and aerospace sectors. The US Air Force Research Laboratory and the National Aeronautics and Space Administration (NASA) employed DT technology to simulate and assess extreme scenarios, testing the resilience of future aerospace flight vehicles against higher loads and more demanding operational conditions [14]. Recognizing its significance, Gartner, a leading global information technology consulting company, has listed DT technology among the top ten strategic trends and emerging technologies for the next 5–10 years [15]. Scholars such as Guo et al. have harnessed DT technology to construct comprehensive DT models spanning the entire lifespan of bearings. They utilized neural networks to obtain dynamic response outcomes from the mechanical model of bearings, thereby uncovering the evolutionary patterns of their life cycles [16]. Piltan et al. combined DT technology with machine learning to detect abnormal bearings and recognize crack sizes [17]. Zhao et al. employed DT technology to establish a model for wind turbine gearboxes, leveraging deep learning networks to accurately classify the operating conditions of these gearboxes [18]. Jahangiri et al. developed a mechanical model of a wind turbine transmission system using a DT approach, enabling the monitoring and identification of changes in structural model parameters for making damage assessments [19]. Moreover, DT technology has recently found application in various fields, including construction [20], medical care [21], and communication [22]. Within the domain of rolling bearing fault diagnosis, DT technology assumes a pivotal role. It facilitates the replication of rolling bearings in the digital realm, generating sample datasets that exhibit the same characteristic distribution. By simulating multidimensional and multi-field high-fidelity twin models, it becomes feasible to emulate bearing conditions under diverse operating circumstances and achieve fault diagnosis. Simultaneously, DT technology presents a new avenue for addressing the challenge of limited sample sizes in rolling bearing fault diagnosis, thus revolutionizing the research pertaining to the identification and diagnosis of bearings in rotating mechanical equipment.

In light of the disparity observed between the feature distributions of training and testing data, certain researchers have incorporated the principles of transfer learning into the realm of bearing fault diagnosis. Transfer learning leverages knowledge acquired from relevant source domains to make predictions in target domains, thereby facilitating a more profound comprehension of feature knowledge in the target domain and enhancing the model’s generalization capabilities. Zhou et al. [23], at the helm of a team of researchers, have introduced a Transfer Learning Residual Network model (TL-ResNet) that combines residual networks and transfer learning techniques. This approach involves the conversion of one-dimensional vibration data into time-frequency images, followed by the transfer of training from the source domain dataset to the target domain bearings, ultimately enabling fault diagnosis in rolling bearings within the target domain. Huang et al. [24] have put forth a profound deep transfer learning model that commences by judiciously selecting a suitable source domain dataset using the maximum mean discrepancy technique to support model training. Domain features are subsequently extracted using specialized domain feature extractors, and the alignment of classifier outputs is achieved via the Wasserstein distance. This approach proves efficacious in diagnosing faults in bearings under diverse operating conditions. Presently, the prevailing method in transfer learning entails constructing a fault diagnosis model employing experimental bench running data as the source domain dataset. However, the dissimilarities in the physical attributes of real working condition main bearings on the experimental bench, coupled with the inherent limitations in simulating operating conditions and environments, significantly impact the accuracy of fault diagnosis outcomes. 

The aim of DT technology is to diminish the dependence on experimental data sets as the source domain by creating high-fidelity twin models and acquiring a comprehensive and balanced sample data set. It also strives to reduce the disparity in data distribution between the source and target domains by incorporating transfer learning into the diagnostic model framework. This integration helps to alleviate errors caused by imbalanced data distribution during the transfer of features and hyperparameters. In the research framework of rolling bearing fault diagnosis based on DT data, the selection of the network for feature extraction holds paramount importance. Wang et al. [25] introduced a multi-scale attention mechanism residual network model (MSA-ResNet) that augments feature sensitivity by integrating attention mechanisms into each residual module. This model employs multi-scale convolution kernels to extract features from non-linear vibration signals and exhibits notable advantages in the accuracy of bearing fault classification. Huang et al. [26] proposed a Channel Attention Mechanism Multi-Scale Convolutional Neural Network (CA-MCNN) model, which enhances the feature learning capabilities of the convolutional layers through the introduction of attention mechanisms. It effectively captures multi-scale information via a one-dimensional convolutional network. Experimental results validate the exceptional fault diagnosis performance of the model across various operating conditions. Zhang et al. proposed a bearing fault detection method based on an improved denoising autoencoder (DAE) and the bottleneck layer self-attention mechanism (MDAE-SAMB) [27]. They achieved high-accuracy online bearing fault classification using only a limited number of fault samples for offline training. Hou et al. presented a bearing fault diagnosis method that combines the Transformer and Residual Neural Network (ResNet) for joint feature extraction [28]. They employed a transfer learning strategy with fine-tuning to alleviate the training challenges of the proposed method in new tasks. The results exhibited superior prediction accuracy in high-noise environments compared to traditional deep learning networks. Zhao et al. proposed a dynamic capsule network with adaptive shared weights (DCCN) and adaptively adjusted convolutional weights using attention mechanisms [29]. The effectiveness of the proposed method was validated through experiments on noisy and variable load-bearing faults, demonstrating a certain degree of generalizability. Wang et al. introduced a dual-stream hybrid generative data-based dual-attention feature fusion network (DAFFN) [30]. They designed a feature fusion network with dual attention mechanisms to learn channel-level and layer-level weights for features. The results demonstrated that the proposed method maintained a certain diagnostic performance even with imbalanced datasets. The research indicates that deep learning networks are extensively employed in the field of bearing fault diagnosis. However, their deep-layered structure may give rise to gradient disappearance or explosion issues, resulting in an inefficient or slow convergence of the network, subsequently reducing the accuracy of bearing fault diagnosis. To tackle this challenge, this article proposes an enhanced ConvNext approach for bearing fault classification. As a next-generation convolutional neural network, ConvNext incorporates exemplary designs from ResNet and Swin Transformer, which have achieved remarkable success in the field of computer vision. Furthermore, the novel architectural design of ConvNext facilitates smoother network gradients, enabling faster convergence. To further enhance the performance of the basic network model, this article enhances the Block module of the ConvNext network by introducing a SimAM attention module after depthwise convolution. This module computes the similarity between two input sequences and fuses their features without introducing additional parameters, thereby improving the overall performance of the basic network. Simultaneously, an ECA attention module is inserted before the Layer Scale to allocate greater attention to fault features and reinforce the directionality of fault feature extraction, thus maximizing the utilization of fault features. Consequently, this paper employs the enhanced ConvNext network to construct a fault recognition model for rolling bearings.

Lastly, this article presents a fault diagnosis model framework for rolling bearings, incorporating DT data, transfer learning, and an enhanced ConvNext network. More specifically, the DT system for the rolling bearing is established by constructing a coupled reduced-order model (ROM) that encompasses the multi-physics field of the main bearing. This model is utilized to enrich the sample dataset of the source domain by introducing different faults and altering various environmental parameters within a specific range. Subsequently, an upgraded version of the ConvNext network model is initially formulated and trained using the source domain dataset. The parameters and model of this improved ConvNext network are then transferred to the rolling bearing through weight and feature transfer. Ultimately, precise and accurate fault recognition of the defective bearing is accomplished through the utilization of the enhanced ConvNext deep learning network. The specific contributions are delineated as follows:

(1) A digital twin system has been devised for rolling bearings, incorporating the integration of multiple physics domains and employing model order reduction techniques. This system facilitates the creation of a substantial and well-balanced dataset, effectively mitigating the challenge posed by limited samples in fault diagnosis. Such an approach not only ensures cost-effectiveness but also enhances convenience.
(2) The ECA-SimAM-ConvNext network model is introduced as an innovative classification framework for detecting rolling bearing faults. This model utilizes the ConvNext convolutional neural network as its foundation and integrates a parameter-free attention module (SimAM) and an efficient channel attention feature module (ECA) at strategic positions. These augmentations significantly enhance the network’s ability to extract fault features, resulting in improved performance.
(3) An innovative methodology is presented for the identification of rolling bearing faults, integrating digital twin data, transfer learning principles, and deep learning algorithms. The efficacy, precision, and superiority of this approach have been substantiated through experimental validation.

The paper is structured into multiple sections, each serving a distinct purpose. Section 2 delves into the discussion of the digital twin system for rolling bearings, encompassing the construction of coupled reduced-order models for the multi-physics field and the establishment of the digital twin model. Furthermore, it provides a fundamental understanding of ConvNext, a key theoretical component. In Section 3, we present the TL-ECA-SimAM-ConvNext method, which is proposed in this study and integrated into the digital twin system, forming a novel framework for fault diagnosis and recognition. The feasibility of the proposed fault diagnosis method is demonstrated in Section 4, where two commonly used bearing datasets are combined. The experimental results are presented and compared with alternative intelligent fault diagnosis approaches. Finally, Section 5 concludes the paper, summarizing the findings and implications.

## 2. Methodology

### 2.1. Construction of Rolling Bearing DT Model

**Figure 1:**
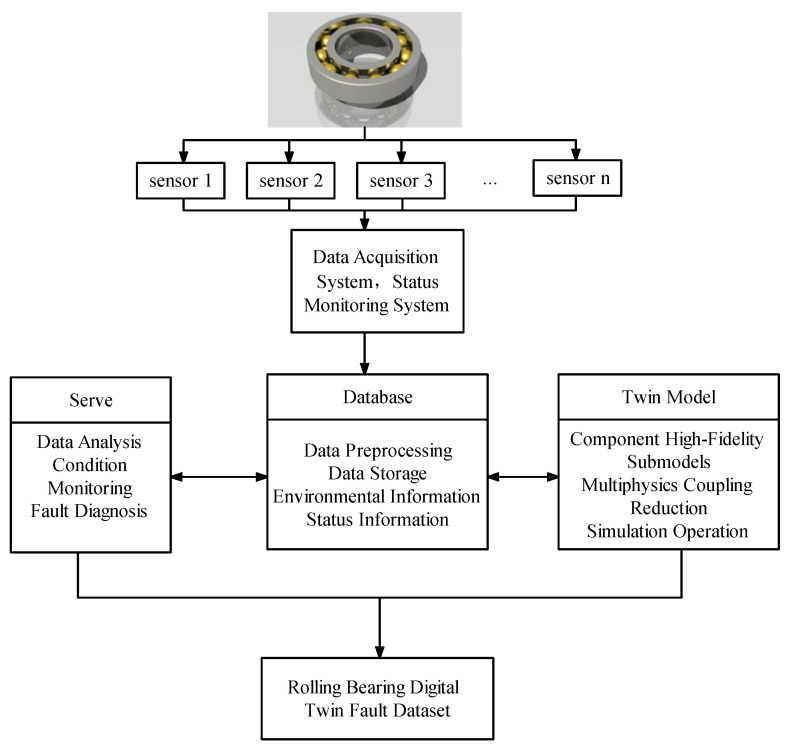
Overall framework of DT system for rolling bearings.

The DT model of the rolling bearing depicted in Figure 1 is introduced in this paper. In this model, the physical entity represents an objective existence that receives instructions and executes specific functions. The twin model has the capability to accurately replicate the physical entity within the digital realm, creating a comprehensive twin model that encompasses multiple dimensions and domains. It facilitates the assessment and surveillance of the physical entity’s reliability. The connection facilitates real-time data interchange between the physical entity and the virtual entity. By analyzing data, it becomes feasible to achieve state monitoring and fault diagnosis of the target entity.

#### 2.1.1. Physical Entity

**Figure 2:**
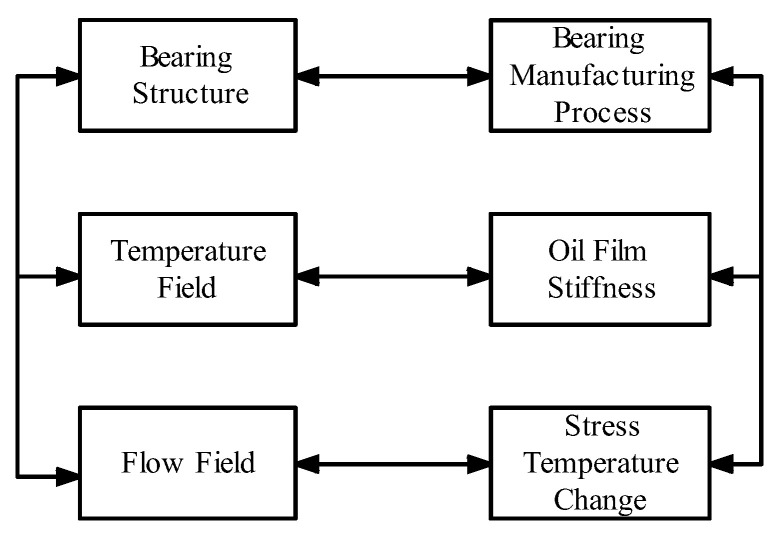
Real working conditions of rolling bearings.

The notion of DT is founded upon the utilization of digital representations to simulate the behavior exhibited by physical entities. In the context of a DT system for rolling bearings, the physical entity serves as the vessel of information, encompassing tangible attributes such as the bearing’s structure, temperature distribution, fluid dynamics, and oil film rigidity. These interconnected attributes exert a mutual influence to ensure the faithful portrayal of the bearing’s performance degradation trend within the virtual model, as exemplified in Figure 2. The construction of a precise twin model platform necessitates the aggregation of diverse operational data and environmental parameters pertaining to the bearing. Communication techniques such as TCP/IP can be employed to establish a connection between the Internet of Things (IoT) platform and sensors embedded within mechanical equipment, thereby enabling seamless data integration. This real-time data acquisition endows the virtual model with efficient and accurate data interchange capabilities that closely resemble the bearing’s actual operational conditions.

#### 2.1.2. Twin Model

The digital twin model precisely maps the physical entity onto the digital realm and mirrors the degradation of rolling bearings by utilizing characteristics derived from historical data. To establish a highly accurate model, this study utilized CAD modeling and CAE finite element simulation software such as SolidWorks and ANSYS. Incorporating factors such as wear, thermal effects, and nonlinear materials, a multi-physics coupled field for the primary bearing was constructed within the ANSYS/Workbench platform. To address the computational time required for prolonged simulations of the complex multi-physics three-dimensional model, which failed to meet real-time demands, the ROM (reduced order model) technique was employed in Ansys Twin Builder, resulting in computational efficiency.

**Figure 3:**
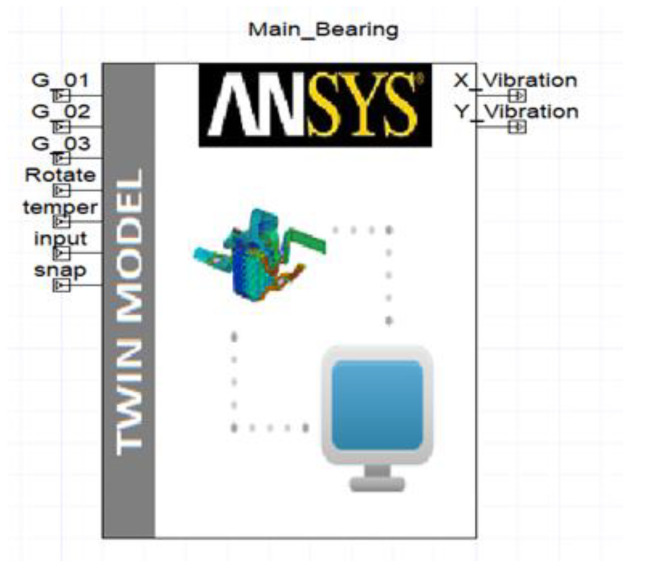
Rolling bearing DT model.

Within the Ansys Twin Builder environment, this study developed a digital twin model for the rolling bearing, as illustrated in Figure 3. Through the meticulous adjustment of virtual sensors and input parameters, vibration displacement signals along the X and Y axes of the rolling bearing were obtained to facilitate the training of the subsequent fault diagnosis model. Ultimately, the encapsulated digital twin model can be seamlessly deployed on IoT platforms such as Microsoft Azure IoT, fostering streamlined connectivity within the digital twin system framework.

### 2.2. ConvNext Network

**Figure 4:**
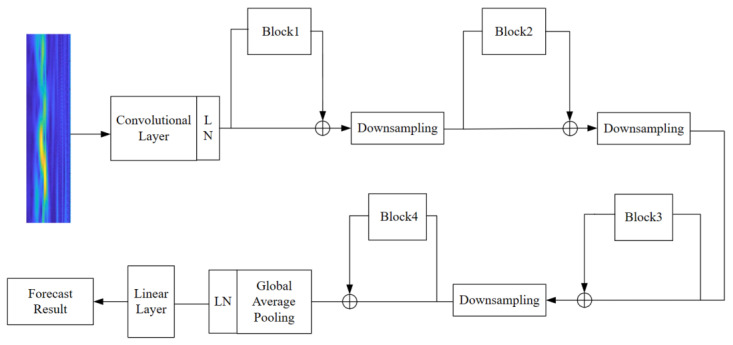
ConvNext Network Structure.

The ConvNext network, introduced by Facebook AI Research (FAIR) in 2022, can be found detailed in reference [31]. This network’s overarching architecture stems from the researchers’ exploration of ResNet and draws inspiration from six key facets of the Swin Transformer network structure, enabling refinements to be made upon this foundation. The comprehensive structure of the ConvNext network is depicted in Figure 4.

**Figure 5:**
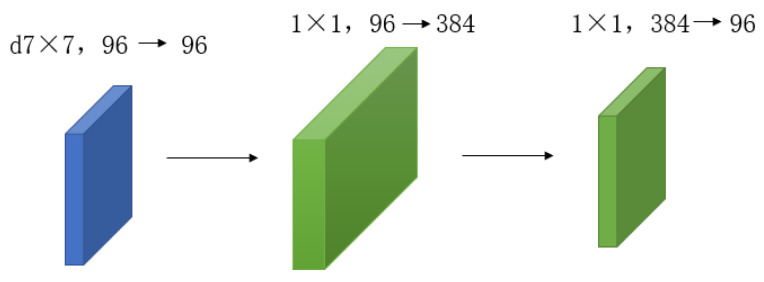
Improvements to ConvNext block module.

In contrast to conventional mainstream network models, the ConvNext network has implemented enhancements across various aspects encompassing the overall structure, deep convolution, inverted bottleneck, large convolution kernel, GELU activation function, and LN layer. Regarding the overall structure, the Stem layer of the ConvNext network employs a convolution kernel of identical size and a four-stride convolution operation akin to the Swin Transformer. As for convolution, the ConvNext network adopts the principle of deep convolution design, segregating the input and output channel quantities to diminish the parameter size of the designed deep convolution, which is significantly smaller than that of traditional convolution. Furthermore, ConvNext incorporates a bottleneck design akin to ResNet. Taking inspiration from the transformer network model, researchers fashioned the block module in ConvNext as an inverted bottleneck structure, resembling that depicted in Figure 5.

**Figure 6:**
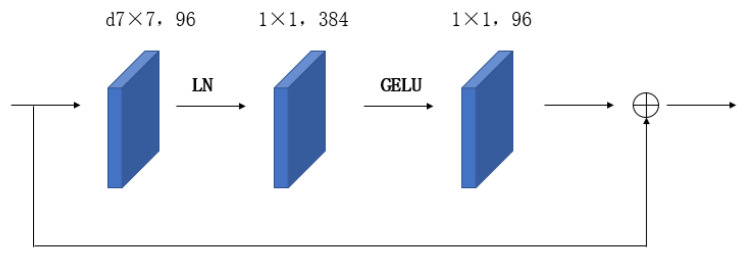
ConvNext Block.

The ConvNext network surpasses traditional neural networks through various advancements. One such improvement involves employing larger 7 × 7 kernels, as opposed to the typical 3 × 3 convolution kernels, to achieve a wider receptive field. Furthermore, ConvNext enhances the activation function by substituting the conventional ReLU activation function with the more effective GELU activation function. Unlike ReLU, which exhibits a drastic gradient change at 0 and lacks the ability to produce negative values, GELU permits negative outputs and possesses a smoother gradient near 0, resulting in faster convergence rates, as depicted in Figure 6.

Moreover, ConvNext replaces the customary BN layer with the LN layer and reduces the number of normalization layers, thereby eliminating redundancy. The LN layer is positioned after the initial convolution layer within each convolution block, as illustrated in Figure 6. These collective improvements augment the ConvNext network’s overall performance and efficiency.

## 3. DT-TL-ECA-SimAM ConvNext Model Bearing Fault Diagnosis Framework

**Figure 7:**
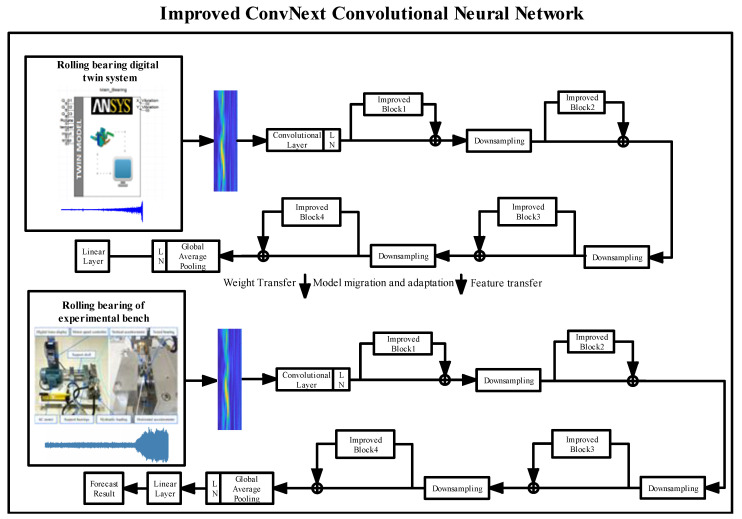
DT-TL-ECA-SimAM-ConvNext model framework.

This paper presents a framework for the fault diagnosis and identification of rolling bearings, as depicted in Figure 7. The proposed approach can be summarized as follows: Step 1: By manipulating the input parameters of the rolling bearing’s X and Y direction vibration displacement signals through virtual sensors within the construction of the digital twin model, source domain datasets of rolling bearing simulation data under various operational conditions are generated. These datasets are then transformed into time-frequency maps using continuous wavelet transform in MATLAB. Subsequently, preliminary training of the ECA-SimAM-ConvNext network model is conducted. Step 2: The ECA-SimAM-ConvNext model is transferred to the target domain rolling bearings through weight and feature migration techniques. Step 3: The DT-TL-ECA-SimAM-ConvNext network model is employed to accomplish precise fault diagnosis and the identification of rolling bearings.

**Figure 8:**
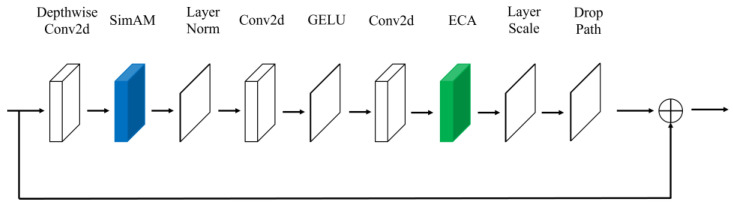
Improved ConvNeXt Block.

This article introduces an enhanced Block module within the ConvNext foundational network, referred to as the ECA-SimAM-ConvNext network model, illustrated in Figure 8. Recent research has shown that the inclusion of ECA and SimAM attention modules within the Block module significantly improves the model’s proficiency in extracting fault features from images. To be precise, the integration of SimAM and ECA attention modules enhances the model’s perception of crucial features, emphasizing essential fault characteristics while suppressing noise. This augmentation strengthens the network’s ability to represent features, thereby facilitating improved differentiation among various bearing states. Through the adaptive selection of frequency ranges or spatial regions of interest, the model can effectively capture signal information related to faults, thus enhancing its adaptability to different types of bearing faults and ultimately boosting generalization performance.

### 3.1. SimAM

**Figure 9:**
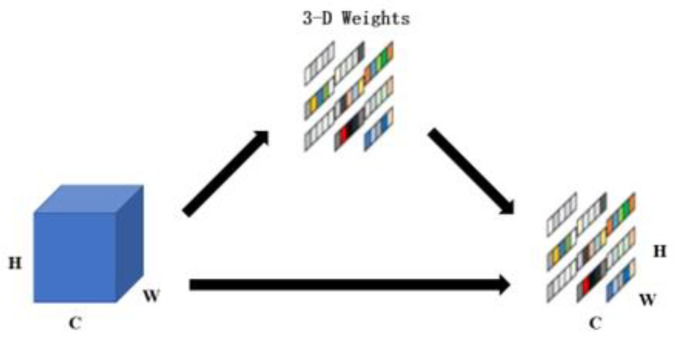
Model structure of SimAM.

Research has unveiled the utilization of attention mechanisms by the human brain to effectively process intricate information. In the realm of deep learning, the integration of attention mechanisms allows for the allocation of varying weights to different segments of input data. This augmentation enhances the model’s interpretive capabilities by enabling a heightened focus on pertinent information while reducing attention towards extraneous details. Drawing inspiration from neuroscience theory, researchers have introduced SimAM [32], an attention module devoid of parameters, as depicted in Figure 9.

The researchers have defined the following energy function by seeking the method of identifying significant neurons, which measures the linear separability between neurons:

(1) $$e_{t}=\left(\omega_{t},b_{t},y,x_{i}\right)={\left(y_{t}-\overset{\wedge }{t}\right)}^{2}+\frac{1}{M-1}{\sum_{i=1}^{M-1}\left(y_{0}-\overset{\wedge }{x_{i}}\right)}^{2}$$

Among them, using binary labels and adding regular terms, the final energy function is defined as follows:

(2) $$e_{t}=\left(\omega_{t},b_{t},y,x_{i}\right)=\frac{1}{M-1}{\sum_{i=1}^{M-1}\left(-1-\left(\omega_{t}x_{i}+b_{t}\right)\right)}^{2}+{\left(1-\left(\omega_{t}t+b_{t}\right)\right)}^{2}+\lambda \omega_{t}^{2}$$

The minimum energy can be obtained by the following formula:

(3) $$e_{t}^{*}=\frac{4\left(\overset{\wedge }{\sigma^{2}}+\lambda \right)}{{\left(t-\overset{\wedge }{u}\right)}^{2}+2\overset{\wedge }{\sigma^{2}}+2\lambda }$$

Among them, t is the target neuron, and μ and $\sigma^{2}$ are the mean and variance of the remaining neurons. It can be seen from Formula (3) that the lower the energy, the greater the difference between neuron t and the surrounding neurons, and the higher the importance. Therefore, the importance of neurons can be obtained by $1/e_{t}^{*}$.

According to the definition of attention mechanism, the features need to be enhanced:

(4) $$\tilde{Χ}=sigmoid\left(\frac{1}{E}\right)⊙X$$

Through the integration of the SimAM module into the network, it becomes feasible to bolster the network’s capacity for feature representation, expedite network convergence, mitigate overfitting to the training data, and consequently amplify the network’s prowess in image recognition.

### 3.2. ECA

**Figure 10:**
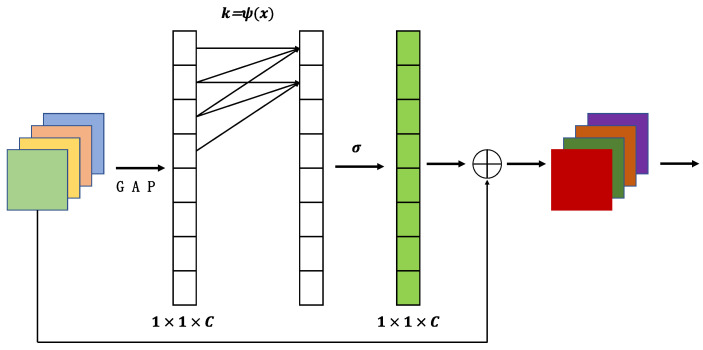
Model structure of ECA.

ECA-Net is a channel attention module that was introduced during the 2020 CVPR conference [33]. It enhances the channel features of the input feature map while preserving its original size. The module is visually represented, and the ECA module model is presented in Figure 10.

The ECA attention module begins by performing global average pooling on the input feature maps, resulting in a 1 × 1 × C feature map. It then learns weights for different channels to enhance the channel features of the input feature map. These channel weights are applied to each channel of the input feature map, and the resulting channel-weighted feature map is obtained through element-wise multiplication. The output feature map, with channel attention, maintains the same size as the original feature map. By incorporating the ECA module into the ConvNext network, significant improvements in model performance can be achieved, while simultaneously reducing model complexity. This module enables the adaptive adjustment of the importance of each channel while eliminating unnecessary information, thereby enhancing the model’s representational capacity to capture key features in the image.

## 4. Experimental Verification

The proposed fault diagnosis method’s feasibility and effectiveness are validated in this section through experimentation on two distinct bearing datasets: the publicly available dataset from Case Western Reserve University and the rolling bearing fault dataset from Xi’an Jiaotong University. Two sets of experiments were conducted to compare the results with mainstream algorithms, employing accuracy and loss functions, confusion matrices, and two-dimensional T-SNE visualization graphs.

The model employed the Adam optimization method to update parameters via backpropagation. It utilized the classic cross-entropy loss function, a batch size of 32, a learning rate of 0.0001, and weight decay set at 0.001.

### 4.1. CWRU Bearing Dataset

**Figure 11:**
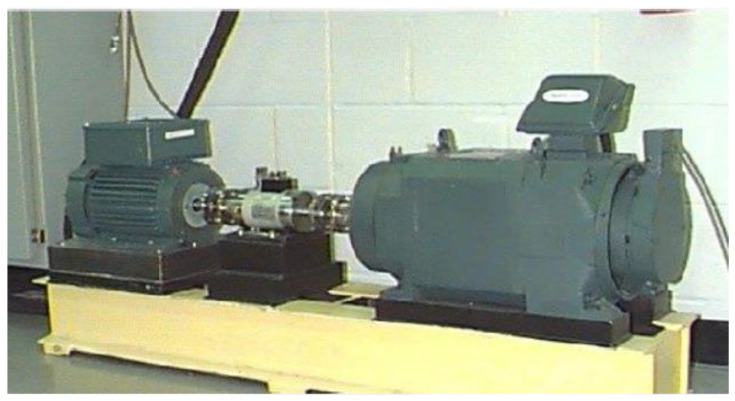
CWRU rolling bearing test platform.

Figure 11 illustrates the experimental setup at Case Western Reserve University (CWRU). The dataset employed in the experiment comprises vibration signals obtained from a SKF-manufactured rolling bearing model 6205-2RS. The signals were collected at a sampling frequency of 12 kHz, encompassing four distinct operational conditions. For each operational condition, experiments were conducted on rolling bearings featuring single-point faults introduced on the ball, inner race, and outer race, with fault diameters measuring 0.18 mm, 0.355 mm, and 0.533 mm, respectively. Additionally, normal rolling bearings were included in the study. Altogether, Table 1 displays a comprehensive overview of ten distinct fault types.

Through the amalgamation of the synthetically produced virtual vibration signals along the X and Y directions, derived from the digital twin model of the rolling bearing, with the authentic vibration signals from the CWRU dataset, employing non-overlapping segmentation, and subjecting each set of data points to continuous wavelet transform to produce relevant time-frequency spectrogram samples, we acquired the total count of experimental samples. The training set encompasses 140 experimental time-frequency images derived from the testing apparatus, as well as 1000 time-frequency images generated through the implementation of the digital twin model, as exemplified in Table 2.

**Figure 12:**
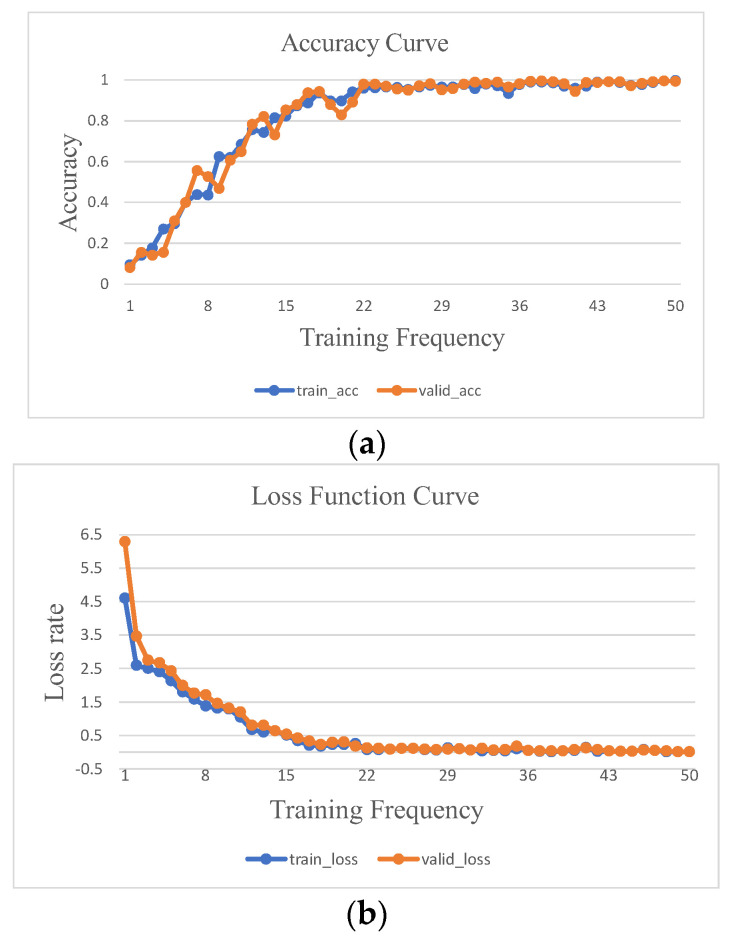
Accuracy and loss rate curve of DT-TL-ECA-SimAM-ConvNext model training. (**a**) Accuracy, (**b**) loss rate.

The accuracy and loss curves depicted in Figure 12 illustrate the outcomes of the DT-TL-ECA-SimAM-ConvNext model after 50 training epochs, utilizing the rolling bearing experimental sample data from Table 2. By the 40th epoch, the accuracy of both the training and validation sets showcased in Figure 12a had reached a flawless 100%, while the loss function value in Figure 12b had descended below the threshold of 0.01. Subsequently, the accuracy and loss curves demonstrated a gradual plateau, indicating the model had achieved a state of stable convergence. These findings conclusively establish that the proposed DT-TL-ECA-SimAM-ConvNext fault diagnosis model exhibited a commendable classification performance when employed with this experimental dataset.

In the domain of machine learning and statistics, a confusion matrix assumes a pivotal role as a tabular representation utilized to assess the efficacy of a classification algorithm. In the context of a classification problem, the confusion matrix enables a comprehensive evaluation of the algorithm’s predictive capabilities by contrasting the predicted categories with the actual categories, thereby elucidating both the accuracy and errors of the classification algorithm. Each row of the confusion matrix corresponds to the actual category, while each column represents the predicted category.

**Figure 13:**
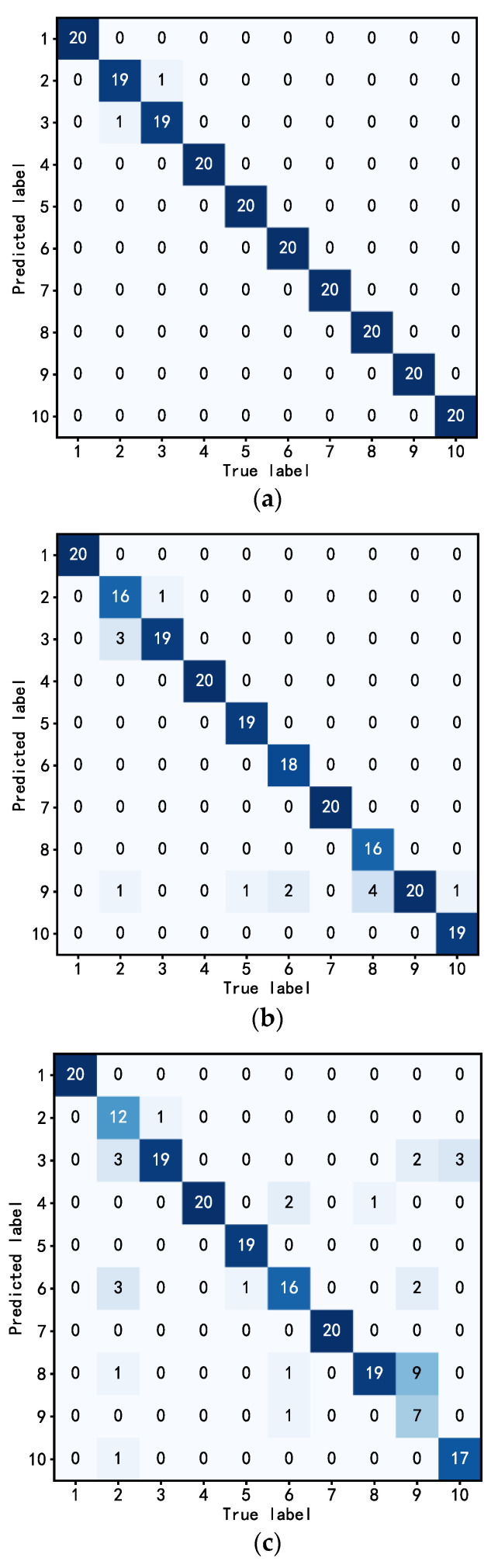
Classification confusion matrix for fault diagnosis of each algorithm. (**a**) DT-TL-ECA-SimAM-ConvNext, (**b**) TL-ConvNext, (**c**) ResNet.

Initially, leveraging the empirical data outlined in Table 2, this investigation conducted a series of replicated experiments to evaluate the proposed approach. The classification outcomes of the test set were visually represented using a confusion matrix, showcased in Figure 13. An analysis of Figure 13a reveals that the proposed method encountered only one instance of mutual misclassification between a 0.355 mm rolling element fault sample and a 0.533 mm rolling element fault sample. Remarkably, the remaining classification results were accurate, and even the misclassifications pertained to minor faults, thereby signifying the presence of a discernible warning effect within the proposed method. A comparison of the experimental findings in Figure 13b,c highlights that the DT-TL-ECA-SimAM-ConvNext model, proposed in this study, achieved superior recognition accuracy in diagnosing diverse types of faults in rolling bearings, surpassing the performance of traditional algorithms.

**Figure 14:**
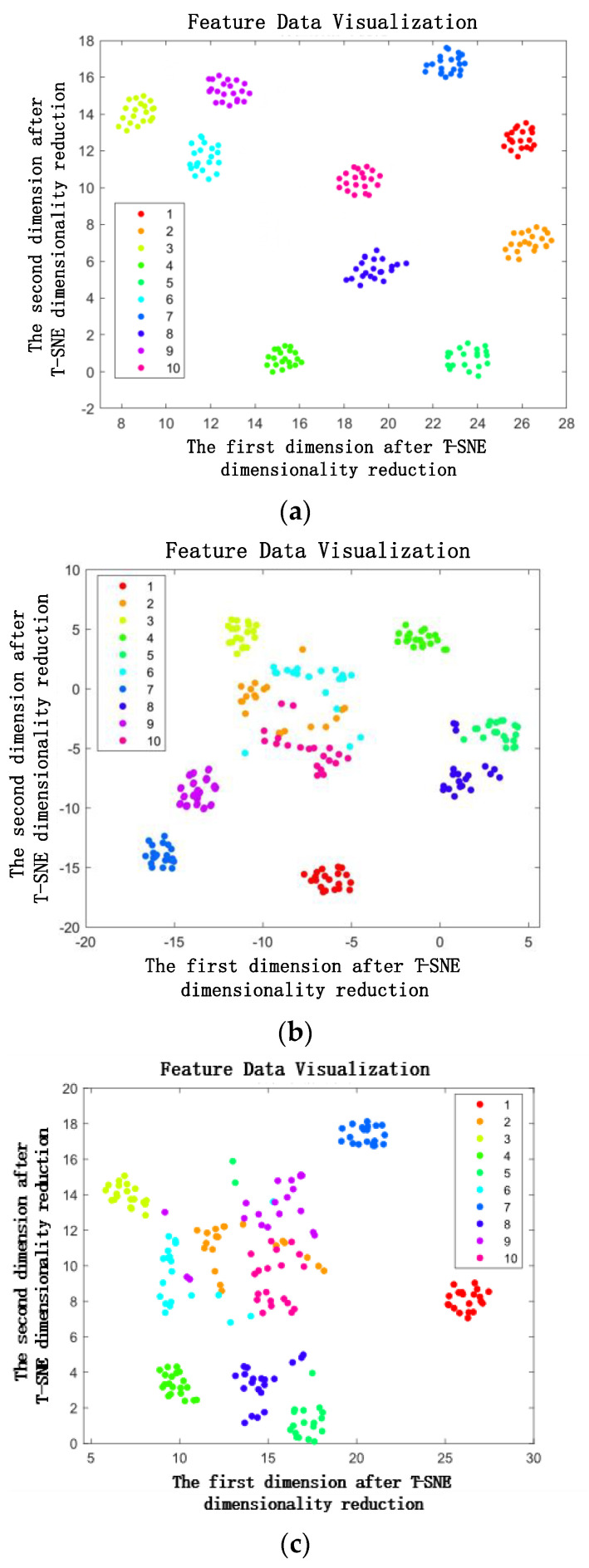
Scatter diagram of fault diagnosis classification characteristics of each algorithm. (**a**) DT-TL-ECA-SimAM-ConvNext, (**b**) TL-ConvNext, (**c**) ResNet.

To present a more visually comprehensive demonstration of the proposed model’s adeptness in feature extraction, t-SNE, a machine learning algorithm employed for nonlinear dimensionality reduction and the visualization of high-dimensional data, was employed. By applying the t-SNE algorithm, the deep learning algorithm employed in this paper effectively reduced the high-dimensional fault features to two dimensions, showcasing them in the form of a scatter plot, as depicted in Figure 14.

The findings depicted in Figure 14 reveal variations in the classification of bearing fault features across different algorithms. Specifically, within this study, the proposed DT-TL-ECA-SimAM-ConvNext fault diagnosis model exhibits remarkable enhancements in the effectiveness and distinctiveness of feature classification. This improvement stems from its adaptive feature extraction approach and dimensionality reduction techniques applied to the test set data, thereby ensuring the absence of overlapping regions between distinct fault types (as exemplified in Figure 14a). In comparison, the TL-ConvNext model, which relies on a traditional experimental bench fault dataset as its source domain for learning (as depicted in Figure 14b), and the ResNet, a classic network model (as demonstrated in Figure 14c), demonstrate a degree of accuracy in certain fault feature classifications. However, they still encounter instances where overlapping regions exist, resulting in ambiguous classification outcomes.

Drawing upon Table 3, along with Figure 13a and Figure 14a, it becomes apparent that the DT-TL-ECA-SimAM-ConvNext fault diagnosis model proposed within this research not only adeptly discriminates the distinguishing features among ten distinct states of rolling bearings within the test set but also sustains a commendable level of accuracy. This serves as a testament to the model’s precision and efficacy.

### 4.2. XJTU-SY Bearing Dataset

In order to showcase the model’s capacity for generalization, this study employs the XJTU-SY bearing dataset [34], sourced from the publicly available experimental dataset of Xi’an Jiaotong University. This comprehensive dataset encompasses vibration signals throughout the complete lifecycle of 15 rolling bearings operating under three distinct conditions, accompanied by explicit labels indicating the positions of failure for each bearing.

**Figure 15:**
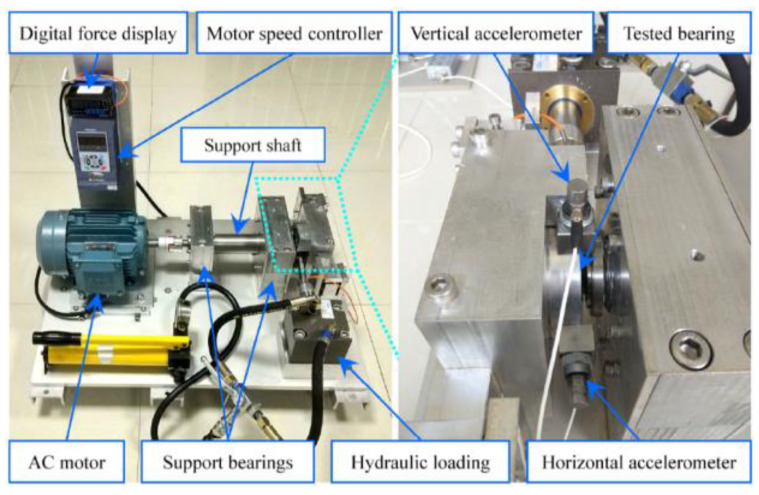
Bearing accelerated life test bench.

Figure 15 depicts the experimental platform of the XJTU-SY bearing dataset, encompassing an AC motor, an electric motor speed controller, a rotating shaft, support bearings, a hydraulic loading system, and the test bearings themselves. This sophisticated platform facilitates accelerated life tests on various rolling or sliding bearings under diverse operating conditions, thereby capturing the full range of vibration signals throughout the lifespan of the test bearings. Notably, the operating conditions of the test platform can be precisely adjusted, primarily in terms of radial force and rotation speed. The hydraulic loading system generates the radial force, exerted upon the bearing seat of the test bearing, while the AC motor’s speed controller establishes and fine-tunes the rotation speed.

The bearings employed for experimentation within this study consisted of LDK UER204 rolling bearings. The experimental design encompassed three distinct operating conditions, as illustrated in Table 4. Each operating condition involved a set of five bearings, with a sampling frequency of 25.6 kHz and a sampling interval of 1 min. Each sampling period lasted for 1.28 s. The specific bearing fault data selected for analysis are presented in Table 5.

In a manner akin to Experiment 1, the virtual X and Y-direction vibration signals, emanating from the rolling bearing digital twin model, are amalgamated with the original vibration signals sourced from the experimental test rig dataset. Employing non-overlapping segmentation, a collection of time-frequency spectrogram samples is derived, generating a comprehensive pool of experimental samples. The training set encompasses 350 experimental time-frequency images from the test rig, along with 1000 time-frequency images engendered by the digital twin model. As delineated in Table 6, the dataset is further partitioned into distinct training, validation, and testing sets.

**Figure 16:**
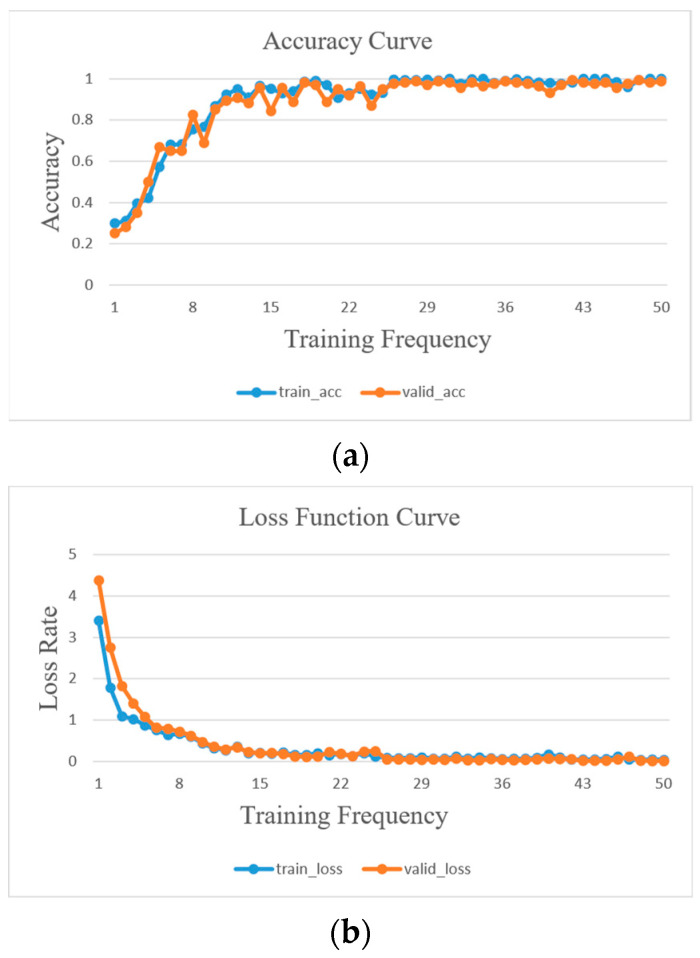
Accuracy and loss rate curve of DT-TL-ECA-SimAM-ConvNext model training. (**a**) Accuracy, (**b**) loss rate.

Upon subjecting the training set data from Table 6 to 50 iterations of the training process using the proposed DT-TL-ECA-SimAM-ConvNext model, the accuracy and loss curves are visualized in Figure 16a and Figure 16b, respectively. Figure 16a exhibits a remarkably stable curve with minimal fluctuations, showcasing the accuracy of the validation set as being slightly below that of the training set. Simultaneously, Figure 16b signifies that the loss rate of the validation set marginally surpasses that of the training set, confirming the absence of overfitting and affirming the satisfactory training effectiveness.

**Figure 17:**
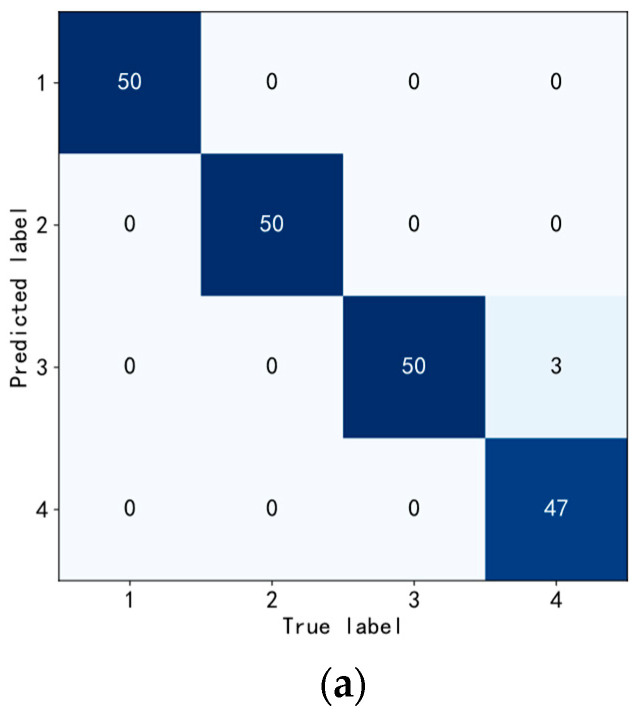

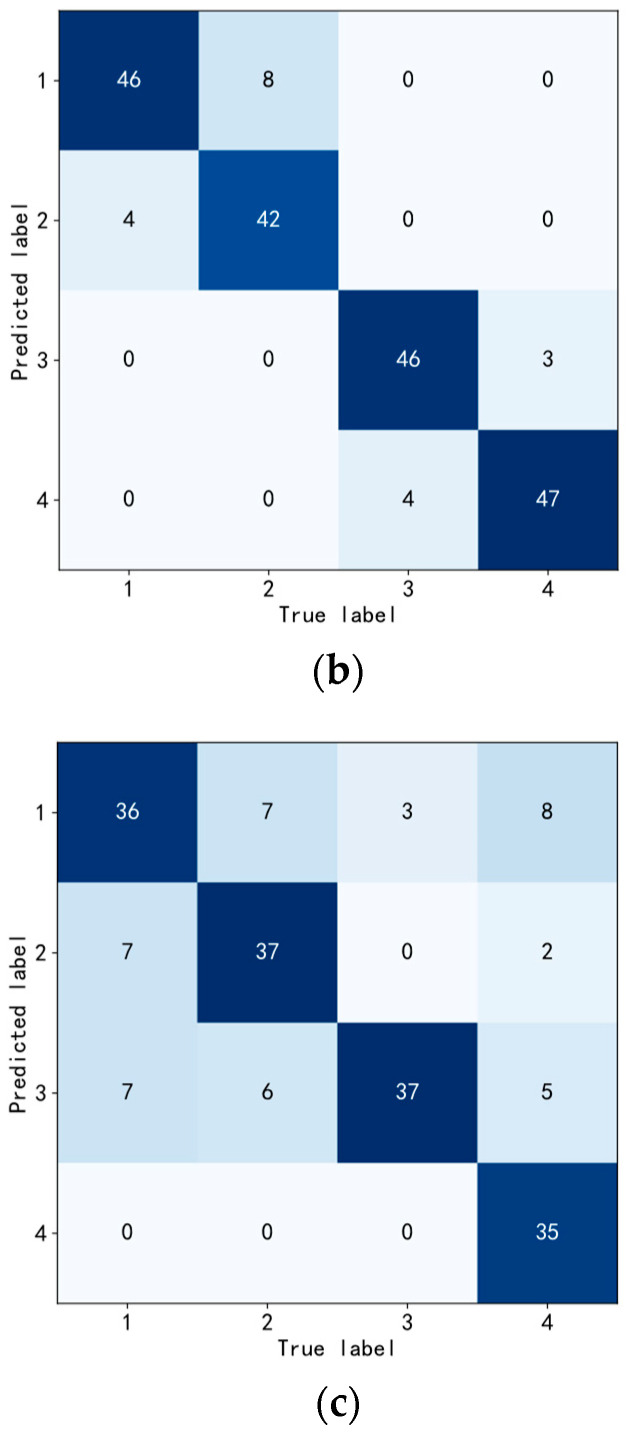
Classification confusion matrix for fault diagnosis of each algorithm. (**a**) DT-TL-ECA-SimAM-ConvNext, (**b**) TL-ConvNext, (**c**) ResNet.

To further evaluate the proficiency of the DT-TL-ECA-SimAM-ConvNext model in discerning bearing faults, a comprehensive analysis was performed utilizing a confusion matrix. This matrix, presented in Figure 17, provides intricate insights into the quantitative assessment of misclassifications among various fault types found in rolling bearings.

Examining Figure 17a, it becomes evident that the proposed DT-TL-ECA-SimAM-ConvNext algorithm outperforms traditional deep learning algorithms in terms of misclassified fault samples. Merely three samples were misjudged, all erroneously classified as outer ring faults for mixed faults. The complexity of real-world bearing operating environments, coupled with intricate dynamic interactions between the inner and outer races, cage, and rolling elements, contributes to the potential misjudgment of mixed fault types. Nonetheless, the model attains remarkable recognition accuracy in other bearing fault categories. Furthermore, the TL-ConvNext approach in this paper employs conventional experimental benches as the source domain dataset, exhibiting commendable classification ability in comparison to the classic ResNet algorithm, as depicted in Figure 17b,c. However, it falls short of the effectiveness achieved by the proposed method in certain fault classifications. This discrepancy arises from the heavy reliance of traditional transfer algorithms on the quality and quantity of the experimental bench dataset. Leveraging both digital twinning and experimentally generated datasets as the source domain dataset, the algorithm captures more intricate fault characteristics, resulting in enhanced precision during subsequent fault classifications while maintaining a certain level of reliable quality and augmenting the number of source domain datasets.

**Figure 18:**
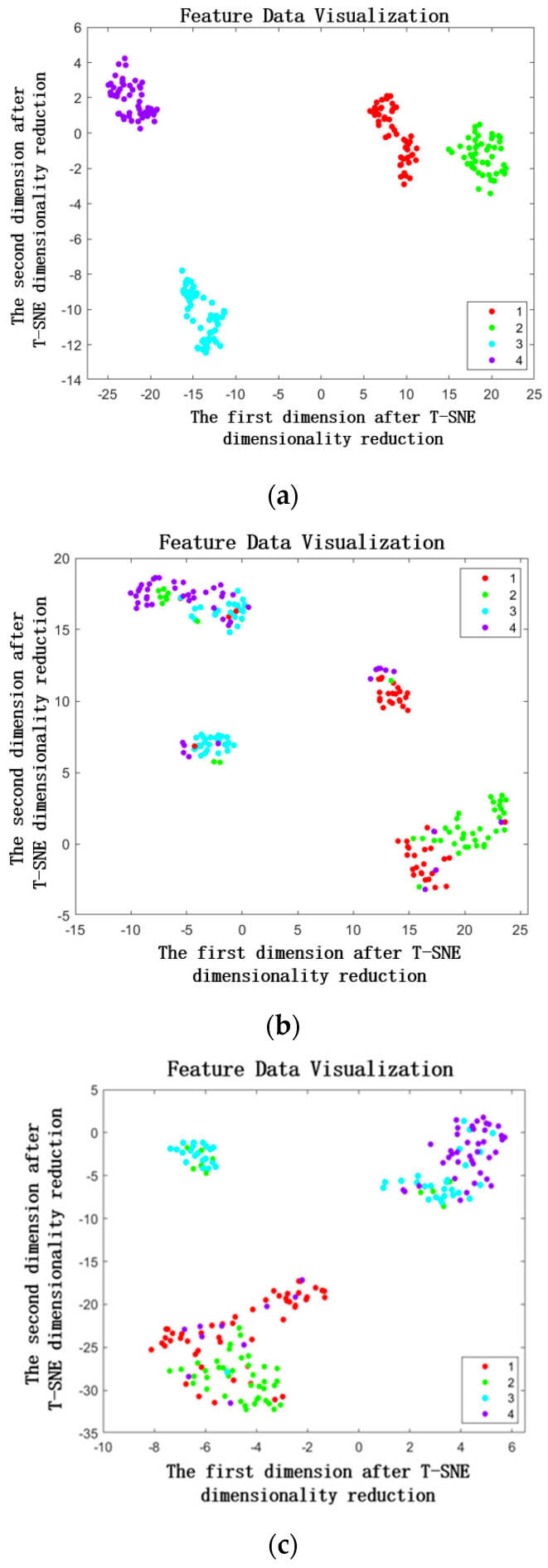
Scatter diagram of fault diagnosis classification characteristics of each algorithm. (**a**) DT-TL-ECA-SimAM-ConvNext, (**b**) TL-ConvNext, (**c**) ResNet.

To visually illustrate the diagnostic prowess of the proposed algorithm, t-distributed stochastic neighbor embedding (t-SNE) analysis was employed to visualize the output outcomes of various algorithms on the XJTU-SY dataset, as showcased in Figure 18.

Through the reduction of high-dimensional data in the test set to a two-dimensional visualization, Figure 18a reveals the remarkable performance of the proposed DT-TL-ECA-SimAM-ConvNext model in accurately classifying bearing fault points, surpassing other conventional deep learning models. This superiority stems from the model’s heightened sensitivity to capture fault features within the image set, enabling a more precise classification of diverse bearing fault types. To validate the effectiveness of the proposed model, a comparative analysis is conducted with the TL-ConvNext algorithm, utilizing testbed data as the source domain dataset, and the ResNet algorithm without transfer learning, as depicted in Figure 18b,c. The results demonstrate that while the traditional deep learning model can classify certain fault points, it still misclassifies numerous others, leading to suboptimal classification accuracy when compared to the employment of the DT-TL-ECA-SimAM-ConvNext model proposed in this paper.

Moreover, based on the accuracy data presented in Table 7, it is evident that the DT-TL-ECA-SimAM-ConvNext model, leveraging digital twin data as the source domain, attains superior accuracy in categorizing various fault types in comparison to conventional deep learning models. This serves as further confirmation of the exemplary performance exhibited by the proposed model in this research.

## 5. Conclusions

To enhance the precision of rolling bearing fault diagnosis in mechanical equipment, this study introduces a fault diagnosis framework, named DT-TL-ECA-SimAM-ConvNext, which integrates digital twin data, transfer learning theory, and deep learning algorithms. Firstly, addressing the limitations of using laboratory data as the source domain dataset in transfer learning, this paper proposes the utilization of a rolling bearing DT system to replicate real-world operating conditions and generate an extensive dataset. This approach enables the synthesis of experimental datasets, thereby overcoming the scarcity of actual fault data in real-world scenarios. Secondly, for the fault diagnosis model, a novel convolutional neural network called ConvNext is adopted. Compared to conventional deep learning algorithms, ConvNext ensures a smoother network gradient and accelerated convergence. Additionally, by incorporating ECA and SimAM attention modules into specific positions of the Block module, the enhanced network can effectively capture intricate fault characteristics across diverse samples. Lastly, the proposed bearing fault classification method is validated through two sets of design experiments. The results demonstrate the versatility of the DT-TL-ECA-SimAM-ConvNext model, which can be applied to different categories of rolling bearings, various environments, operating conditions, and laboratory settings, thereby serving as a valuable tool for fault diagnosis in rotating mechanical equipment.

## Figures and Tables

**Table 1:** CWRU bearing fault classification and tag value.

Label	Fault Type	Fault Diameter
1	Rolling Element Fault	0.18
2	Rolling Element Fault	0.355
3	Rolling Element Fault	0.533
4	Inner Ring Failure	0.18
5	Inner Ring Failure	0.355
6	Inner Ring Failure	0.533
7	Outer Ring Fault	0.18
8	Outer Ring Fault	0.355
9	Outer Ring Fault	0.533
10	Normal	0

**Table 2:** Number of experimental samples.

Fault Type	Training Set	Verification Set	Test Set
1	140 + 1000	40	20
2	140 + 1000	40	20
3	140 + 1000	40	20
4	140 + 1000	40	20
5	140 + 1000	40	20
6	140 + 1000	40	20
7	140 + 1000	40	20
8	140 + 1000	40	20
9	140 + 1000	40	20
10	140 + 1000	40	20

**Table 3:** Accuracy of each model.

Model	Test Set Samples	Accuracy
LeNet	200	0.793
CNN	200	0.854
ResNet	200	0.885
ConvNext	200	0.925
TL-ConvNext	200	0.965
DT-TL-ECA-SimAM-ConvNext	200	0.998

**Table 4:** Bearing accelerated life test conditions.

Condition Number	1	2	3
Speed/(r/min)	2100	2250	2400
Radial Force/KN	12	11	10

**Table 5:** List of bearing fault data set information selected in this paper.

Label	Failure Location	Data Set	Total Number of Samples
1	Holder	Bearing 2_3	533
2	Inner Circle	Bearing 2_1	491
3	Outer Circle	Bearing 1_1	123
4	Inner Circle, Rolling Element, Cage, Outer Circle (Mixed Fault)	Bearing 3_2	2496

**Table 6:** Number of experimental samples.

Fault Type	Training Set	Verification Set	Test Set
1	350 + 1000	100	50
2	350 + 1000	100	50
3	350 + 1000	100	50
4	350 + 1000	100	50

**Table 7:** Accuracy of each model.

Model	Test Set Samples	Accuracy
LeNet	200	0.786
CNN	200	0.804
ResNet	200	0.825
ConvNext	200	0.903
TL-ConvNext	200	0.946
DT-TL-ECA-SimAM-ConvNext	200	0.995

## Data Availability

Not applicable.
